# Effective artifact removal in resting state fMRI data improves detection of DMN functional connectivity alteration in Alzheimer's disease

**DOI:** 10.3389/fnhum.2015.00449

**Published:** 2015-08-11

**Authors:** Ludovica Griffanti, Ottavia Dipasquale, Maria M. Laganà, Raffaello Nemni, Mario Clerici, Stephen M. Smith, Giuseppe Baselli, Francesca Baglio

**Affiliations:** ^1^IRCCS, Fondazione Don Carlo GnocchiMilan, Italy; ^2^Department of Electronics, Information and Bioengineering, Politecnico di MilanoMilan, Italy; ^3^Nuffield Department of Clinical Neurosciences, Oxford Centre for Functional MRI of the Brain, University of OxfordOxford, UK; ^4^Physiopatholgy Department, Università degli Studi di MilanoMilan, Italy

**Keywords:** functional magnetic resonance imaging, resting state, artifacts, functional connectivity, default mode network, Alzheimer's disease

## Abstract

Artifact removal from resting state fMRI data is an essential step for a better identification of the resting state networks and the evaluation of their functional connectivity (FC), especially in pathological conditions. There is growing interest in the development of cleaning procedures, especially those not requiring external recordings (data-driven), which are able to remove multiple sources of artifacts. It is important that only inter-subject variability due to the artifacts is removed, preserving the between-subject variability of interest—crucial in clinical applications using clinical scanners to discriminate different pathologies and monitor their staging. In Alzheimer's disease (AD) patients, decreased FC is usually observed in the posterior cingulate cortex within the default mode network (DMN), and this is becoming a possible biomarker for AD. The aim of this study was to compare four different data-driven cleaning procedures (regression of motion parameters; regression of motion parameters, mean white matter and cerebrospinal fluid signal; FMRIB's ICA-based Xnoiseifier—FIX—cleanup with soft and aggressive options) on data acquired at 1.5 T. The approaches were compared using data from 20 elderly healthy subjects and 21 AD patients in a mild stage, in terms of their impact on within-group consistency in FC and ability to detect the typical FC alteration of the DMN in AD patients. Despite an increased within-group consistency across subjects after applying any of the cleaning approaches, only after cleaning with FIX the expected DMN FC alteration in AD was detectable. Our study validates the efficacy of artifact removal even in a relatively small clinical population, and supports the importance of cleaning fMRI data for sensitive detection of FC alterations in a clinical environment.

## Introduction

Resting state functional magnetic resonance imaging (rfMRI) is a non-invasive powerful technique for mapping brain function and it is now widely used in both healthy subjects and diseased populations (Deco et al., 2011; Power et al., 2014). rfMRI data, however, are corrupted by many sources of temporal fluctuation (e.g., head movement, physiological noise, scanner instabilities, etc.), which affect the results of functional connectivity (FC) analyses and their interpretation. Although present also in task-induced fMRI, the problem of artifact identification and removal is more difficult in rfMRI because, due to the absence of a priori hypothesis of activation and externally triggered temporal references, it is hard to distinguish the signal related to neural activity from non-neural sources of noise, when the latter are (spatially or temporally) correlated. Moreover, the artifacts may share some spatial or spectral overlap with the resting state networks (RSNs) and affect their correct identification and quantification of their connectivity.

Therefore, there is growing interest in the development of cleaning procedures, especially those not requiring external recordings (data-driven) and which are able to remove multiple sources of artifacts: temporal filtering, regression of motion parameters, regression of mean white matter and cerebrospinal fluid (CSF) signal, scrubbing, multi-echo acquisition sequences, ICA-based techniques, just to mention a few (see Murphy et al., 2013 for a detailed review).

Studies evaluating the efficacy of cleaning procedures for fMRI data are often performed on one group of healthy controls (Tohka et al., 2008; Weissenbacher et al., 2009; Bright and Murphy, 2013; Marx et al., 2013) or on two groups of healthy subjects differing in the amount of a specific artifact, typically head motion (Van Dijk et al., 2012; Satterthwaite et al., 2013). The performance of the clean-up is generally tested in terms of increased within-group consistency of activations and FC maps, reduction of correlation with noise, and decrease of artifact-driven between-group differences. However, it is more difficult to evaluate the success of cleaning when multiple sources of artifacts (not only motion) are removed. As a result of the cleaning procedure, only the inter-subject variability due to the artifacts should be removed, preserving valuable individual differences. In fact, the ability to capture between-subject variability in FC is very important in clinical applications, in order to discriminate different pathologies and monitor their evolution and staging. Moreover, cleaning techniques are usually tested on very good quality datasets (e.g., Griffanti et al., 2014), acquired for research purpose only. However, given that the final aim of clinical research is translation into clinical practice, it is crucial to test those approaches also on images acquired using clinical scanners (which are still predominantly 1.5 T), to test whether the methods used in research are suitable for translation in the clinical environment.

In fact rfMRI, though used only in research so far, is one of the imaging instruments with the highest potential as a new biomarker for neurodegenerative diseases (Greicius et al., 2004; Gili et al., 2011; Li and Wahlund, 2011; Li et al., 2014; Szewczyk-Krolikowski et al., 2014), since it is able to detect subtle functional abnormalities in brain networks supporting complex cognitive processes that are progressively impaired over the course of neurodegenerative pathologies (Brier et al., 2012; Damoiseaux et al., 2012). Alzheimer's disease (AD) is a common neurodegenerative disease related to the aging process. AD is characterized by the presence of widespread functional disturbances in the brain and a decreased FC of the DMN has been consistently observed through rfMRI in its posterior portion (precuneus, posterior cingulate cortex) to the anterior portion (anterior cingulate and medial prefrontal cortex) also involving medial temporal lobe structures (Gili et al., 2011; Brier et al., 2012; Hafkemeijer et al., 2012). Changes in functional connectivity of regions within the DMN have been found also in individuals at high risk for developing AD (Filippini et al., 2009; Sorg et al., 2009; Gili et al., 2011; Hafkemeijer et al., 2012; Cha et al., 2013; Esposito et al., 2013; Wang et al., 2013; Li et al., 2014). In this framework, an effective preprocessing of rfMRI data is crucial, in order to allow the correct identification of this FC alteration.

The aim of this study was to compare four data-driven cleaning procedures (i.e., without the need for external recordings of physiological signals) on data relative to elderly healthy controls (HC) and AD patients in a mild stage of the disease, and to evaluate the impact of the cleaning step on the ability to detect the typical DMN functional connectivity alterations in AD. This because of two reasons: first, since there is no ground truth for signal and noise in fMRI data, due to the great amount of different sources that contribute to the measured signal, we chose such a strong clinical finding as ground truth for “correct” functional connectivity estimation to compare the cleaning methods (keeping constant the dataset and all other processing steps that might have contributed to the results in previous studies); second, given the promising role of rfMRI as a biomarker in AD, we wanted to test the performance of the cleaning approaches on data acquired using a 1.5 T clinical scanner, as a test for possible translation into clinical practice.

In particular, the first two cleaning approaches are commonly used in the preprocessing of rfMRI data: the regression of motion parameters (Satterthwaite et al., 2013) and the regression of motion parameters, mean white matter (WM) signal and mean cerebrospinal fluid (CSF) signal (Fox et al., 2005; Satterthwaite et al., 2013). We did not include global signal regression in our study because it has been demonstrated (Murphy et al., 2009; Saad et al., 2012) that the global regression process introduces anti-correlations that are difficult to interpret. Some studies (Popa et al., 2009; de Pasquale et al., 2010) indicate that the global signal can include a significant amount of neural activity; therefore, many argue that its removal should be avoided. As BOLD signal related to neural activity should be predominantly in the gray matter, we chose as alternative method to regress out of the time series derived from just the WM and CSF voxels (Weissenbacher et al., 2009). The other two methods evaluated in this study are two options of a recently developed ICA-based denoising method, FMRIB's ICA-based Xnoiseifier (FIX) (Griffanti et al., 2014; Salimi-Khorshidi et al., 2014), based on single-subject ICA decomposition followed by automatic classification through hierarchical fusion of classifiers and removal of the contribution of the motion parameters and the full (with the *aggressive* option) or unique (with the *soft* option) variance of the noise components identified by the classifier (see Griffanti et al., 2014 for the mathematical details about the two approaches).

The denoising procedures were firstly compared, separately for the HC and AD groups, in terms of temporal signal to noise ratio (SNR) and BOLD signal fluctuation reductions with respect to the uncleaned data. With the datasets obtained with the different cleaning options, we then performed a FC analysis of the DMN using two methods: seed-based correlation (with the seed located in the posterior cingulate cortex, PCC) and template-based dual regression (Khalili-Mahani et al., 2012, 2013). These are the two main approaches used to investigate FC and the DMN alteration in AD has been found with both (Binnewijzend et al., 2012; Brier et al., 2012; Damoiseaux et al., 2012), so we wanted to evaluate if and how the cleaning approaches affect the two methods. Finally, we compared the FC results in terms of within-group consistency across subjects and pattern of between-group differences, hypothesizing that a more effective cleaning approach would lead to more consistent FC results and would allow a better identification of the well-known pattern of DMN FC alterations in AD patients.

To the best of our knowledge, this study represents the first systematic evaluation of the effect of data-driven (especially ICA-based) noise removal in data acquired from a diseased population at 1.5 T. The clinical validation of the efficacy of this analysis step in a relatively small sample, and therefore a limited statistical power, gives confidence to obtain reliable results, very important for the definition of imaging biomarkers using rfMRI.

## Materials and methods

### Subjects and MRI data acquisition

Data from 41 subjects (20 healthy controls, HC and 21 AD patients) were acquired at Don Gnocchi Foundation, IRCCS Santa Maria Nascente (Milan, Italy) and their characteristics are reported in Table 1. AD patients were recruited from the Memory Clinic of Don Gnocchi Foundation, with a diagnosis of probable AD dementia according to the revised NINCDS-ADRDA criteria (Mckhann et al., 2011) in a mild stage (Clinical Dementia Rating Scale, CDR ≤ 2). The 20 age-matched HC (Mini-Mental State Examination, MMSE ≥ 28) had no history of neurological, cardiovascular or metabolic disorders and voluntarily participated in the study. According to the recommendations of the declaration of Helsinki for investigations on human subjects, both local ethics committee approval of the Don Gnocchi Foundation and written informed consent from all subjects or their caregivers to participate in the study were obtained before study initiation.

**Table 1:** **Subjects' characteristics**.

	**Healthy controls (HC)**	**AD patients**	**Group comparison (*p*-value)^#^**
*N*	20	21	
Age (years)	71.05 ± 3.66	73.62 ± 5.22	n.s. (0.08)
Gender (F:M)	13:7	13:8	n.s. (0.21)
MMSE	29.55 ± 0.69	21.62 ± 2.71	< 0.01
Motion during fMRI acquisition (§)	0.07 ± 0.04	0.09 ± 0.06	n.s. (0.27)

*MMSE, Mini Mental State Examination; (§) mean relative displacement in mm as calculated during the preprocessing with MELODIC FSL tool*.

# *Calculated with two-sample independent t-test or Fisher's exact test, as appropriate*.

*n.s. = not significant*.

MRI acquisitions were performed using a 1.5 T Siemens Magnetom Avanto (Erlangen, Germany) scanner with an eight-channel head coil. Resting state fMRI, BOLD EPI images (TR/TE = 2500/30 ms; resolution = 3.1 × 3.1 × 2.5 mm^3^; matrix size = 64 × 64; number of axial slices = 39; number of volumes = 160; acquisition time 6 min and 40 s) were collected at rest. Subjects were instructed to keep their eyes closed, not to think about anything in particular, and not to fall asleep. T1-weighted 3D scans were also acquired (TR/TE = 1900/3.37 ms; resolution = 1 × 1 × 1 mm^3^; matrix size = 192 × 256; number of axial slices = 176) and used as anatomical references for fMRI analysis and for voxel-based morphometry (VBM) analysis. T2-weighted dual-echo turbo spin echo (TR/TE = 2920/22 ms, FoV = 240 × 180 mm, resolution = 0.75 × 0.75 × 4 mm^3^, number of axial slices = 25) and FLAIR (TR/TE = 9000/121 ms, FoV = 240 × 168 mm, in-plane resolution = 0.94 × 0.94 × 5 mm^3^, number of coronal slices = 24) images were also acquired to limit the risk of including subjects with concomitant vascular pathology (exclusion criteria: one or more macroscopic T2-weighted abnormalities located in the deep white matter (WM) or more than five abnormalities, maximum diameter < 5 mm, located in periventricular regions).

### Voxel-based morphometry (VBM) analysis

In order to verify the typical pattern of atrophy in AD patients, we evaluated gray matter (GM) volume differences between HC and AD. Structural data were analyzed with FSL-VBM (Douaud et al., 2007), an optimized VBM protocol (Good et al., 2001) carried out with FSL. First, structural images were brain-extracted and gray matter-segmented before being registered to the MNI 152 standard space using non-linear registration. The resulting images were averaged and flipped along the x-axis to create a left-right symmetric (to ensure no bias due to possible cerebral asymmetries), study-specific gray matter template. Second, all native gray matter images were non-linearly registered to this study-specific template and modulated to correct for local expansion (or contraction) due to the non-linear component of the spatial transformation. The modulated gray matter images were then smoothed with an isotropic Gaussian kernel with a sigma of 3 mm (corresponding to a full width at half maximum—FWHM—of 7.06 mm). Finally, voxel-wise GLM was applied using permutation-based non-parametric testing, correcting for multiple comparisons across space using the threshold free cluster enhancement (TFCE) approach.

### rfMRI data preprocessing and cleaning approaches

The individual common preprocessing steps for the analysis of rfMRI data were carried out using FSL (Smith et al., 2004; Jenkinson et al., 2012). Firstly, images were motion corrected with MCFLIRT; from this operation the six rigid-body parameter time series were extracted for each subject (to be used for subsequent cleaning) and the mean relative displacement was calculated to ensure that the two groups were matched in terms of average amount of head motion (see Table 1). Non-brain tissues were removed with brain extraction tool (BET), data were spatially smoothed with a 5 mm FWHM Gaussian kernel, and high-pass temporal filtering was applied with a cut-off frequency of 0.01 Hz to remove slow drifts.

Five datasets were obtained with different data-driven cleaning approaches:

1. Uncleaned dataset: only common preprocessing;
2. Dataset obtained with MOTreg approach (Satterthwaite et al., 2013): a regression of 24 motion parameters. (the six rigid-body parameter time series, their backward-looking temporal derivatives, and the squares of the 12 resulting regressors);
3. Dataset obtained with MWCreg approach (Fox et al., 2005; Satterthwaite et al., 2013): regression of 24 motion parameters, WM mean signal and CSF mean signal. Specifically, the WM and CSF mean signal was extracted as the mean time series from each 4D pre-processed dataset within a ventricular region of interest and a region centered in the WM identified in the MNI space and registered to each subject's individual space;
4. Dataset obtained with FIXsoft approach (Griffanti et al., 2014; Salimi-Khorshidi et al., 2014): single-subject spatial ICA with MELODIC (Beckmann and Smith, 2004) with automatic dimensionality estimation followed by ICA-based automatic denoising using FMRIB's ICA-based Xnoiseifier (FIX) removing the full variance of the 24 motion parameters, but only the unique variance of the noisy components (*soft clean-up*);
5. Dataset obtained with FIXagg approach (Griffanti et al., 2014; Salimi-Khorshidi et al., 2014): single-subject spatial ICA with MELODIC (Beckmann and Smith, 2004) with automatic dimensionality estimation followed by ICA-based automatic denoising using FIX. The single-subject components removed with this approach are the same as with FIXsoft, but in this case the full variance of both the 24 motion parameters and of the noisy components is removed (*aggressive clean-up*; see (Griffanti et al., 2014) for the mathematical details about the two approaches).

The training dataset used to clean the data was the same for both groups and it was built with data from healthy controls following the criteria for manual classification of single-subject independent components that are well-established in literature (Kelly et al., 2010; Griffanti et al., 2014; Salimi-Khorshidi et al., 2014). However, due to the modest number of subjects, we were able to manually check that FIX successfully identified the artifactual components also on AD patients' data.

### Measures of bold signal variation

To test how the different cleaning approaches affect the BOLD signal variation in the two groups, we calculated the following measures: a global measure of signal to noise ratio (raw temporal-SNR) and a voxel-wise measure (%ΔSTD_map_) to examine the regional impact of each correction method on the BOLD signal (Khalili-Mahani et al., 2013).

For each dataset, a raw temporal-SNR image was formed dividing the mean image across time by the standard deviation image over time (STDimg). The temporal-SNR image was then eroded to exclude brain-edge effects, and the median SNR-value was calculated and compared between the two groups at each cleaning step and within groups among different cleaning options.

The %ΔSTD_map_ was defined by Khalili-Mahani et al. (2013) as the percentage of the voxel-wise temporal fluctuation amplitude (STDimg) of the uncleaned data that is suppressed by the cleaning. This map was calculated for each subject as the difference between the STDimg of the cleaned datasets and the STDimg of the uncleaned data, with respect to the STDimg of the uncleaned data (Equation 1):

(1) $$\begin{array}{l} \%\Delta {STD}_{map}=\frac{STD\left(img_{uncl}–img_{clean}\right)}{STD\left(img_{uncl}\right)}\cdot 100\% \end{array}$$

The %ΔSTD_map_ of all subjects were then registered to the individual's structural scan using Brain-Boundary Registration (BBR; Greve and Fischl, 2009) and to the 2 mm MNI152 standard space using non-linear registration (FNIRT), and used to build, for each group, a probability map of areas where %ΔSTD > 25% across subjects.

### Functional connectivity analyses

After the cleaning procedures each single subject 4D pre-processed dataset was coregistered to the individual's structural scan using BBR and to standard space using FNIRT, and resampled to 2 × 2 × 2 mm^3^ resolution in the MNI152 space. We then performed DMN functional connectivity analyses with two methods: seed based correlation and template-based dual-regression.

For seed-based correlation analysis, a region of interest (ROI) in the PCC was selected in the MNI152 template (6-mm radius sphere, centered in *x* = 0; *y* = −26; *z* = 52) according to previous studies (Andrews-Hanna et al., 2007; Van Dijk et al., 2010), and the corresponding mean time series was extracted from each 4D pre-processed dataset. Seed-based voxel-wise FC maps were then obtained by computing the linear correlation between the PCC-time series and the time series of all acquired voxels (REST toolbox; Song et al., 2011). Correlation maps were then converted to z-maps using Fisher's r-to-z transformation before entering the statistical analysis.

Template-based dual regression (Khalili-Mahani et al., 2012, 2013) consisted in estimating the FC in terms of fitting the BOLD fluctuations at each voxel with respect to the dominant fluctuation within 10 major RSNs (Smith et al., 2009), which are frequently detected in resting-state functional connectivity analyses (in order to be able to compare the five datasets). Therefore, the 10-RSNs template was used in the first stage of dual regression (Filippini et al., 2009) as a set of spatial regressors to generate individual temporal dynamics and spatial maps of the RSNs of the five datasets. The component corresponding to the DMN of each subject was entered into the statistical analysis.

### Statistical analysis

In absence of a ground-truth of the neural signal, the cleaning of the data should both enhance reproducibility (stability of the functional connectivity measures across subjects) and also discriminability regarding classifications of interest (in this case healthy subjects vs. AD patients). For this reason we tested, for each cleaning procedure, both within-group consistency across subjects of the FC measures and between-group differences.

The within-group consistency of the DMN connectivity for each voxel was measured as the standard deviation of the z-maps (obtained with seed-based correlation or template-based ICA) across subjects, separately for HC and AD groups. The standard deviation maps were then compared voxel-by-voxel within the brain with a paired *t*-test, both across cleaning approaches and between groups.

The comparison between the two groups for the different cleaning approaches was performed on the z-maps obtained with the two methods through a regions-of-interest (ROI) analysis and a voxel-wise whole brain analysis. Mean parameter estimates (P.E.) were extracted from the subject-specific spatial maps within a ROI including the PCC and Precuneum as identified from the Harvard–Oxford atlas (probability threshold 25%), and the obtained values were then compared between the two groups with a two-sample independent *t*-test (results were considered significant at *p* < 0.05). Voxel-wise differences were tested using a non-parametric permutation test (Winkler et al., 2014). Normalized gray matter volume was included as a covariate to control for the effect of atrophy. Multiple comparison correction was applied using cluster-based correction as implemented in FSL. Briefly, it consists of two stages: first, a voxel-level primary threshold defines clusters by retaining groups of supra-threshold voxels (in our case corresponding to *p*_uncorr_ < 0.05). Second, a cluster-level extent threshold, measured in units of contiguous voxels, is determined based on the estimated distribution of cluster sizes under the null hypothesis of no activation in any voxel in that cluster. Results were considered significant for *p*_corr_ < 0.05 within a mean mask created by averaging the gray matter segmentations obtained from each subject's T1-weighted images with FSL–FAST (Zhang et al., 2001) and thresholding it so that only voxels with GM probability of 20% across subjects were included in the analysis.

## Results

### VBM results

Results of the VBM analysis on structural MRI data showed that patients with AD were significantly more atrophic than controls in the medial temporal lobe structures (bilateral hippocampus and parahippocampus), and in several other brain regions including medial, anterior and postero-inferior regions of temporal lobes bilaterally, precuneus/posterior cingulate, thalamus, basal ganglia (putamen and caudate nuclei), and frontal lobes. These results (see Supplementary Figure 1) are consistent with the well-known pattern of gray matter atrophy typical of AD, as described in several previous studies (Busatto et al., 2008; Zamboni et al., 2013).

### Effect of cleaning on bold signal variation

**Figure 1:**
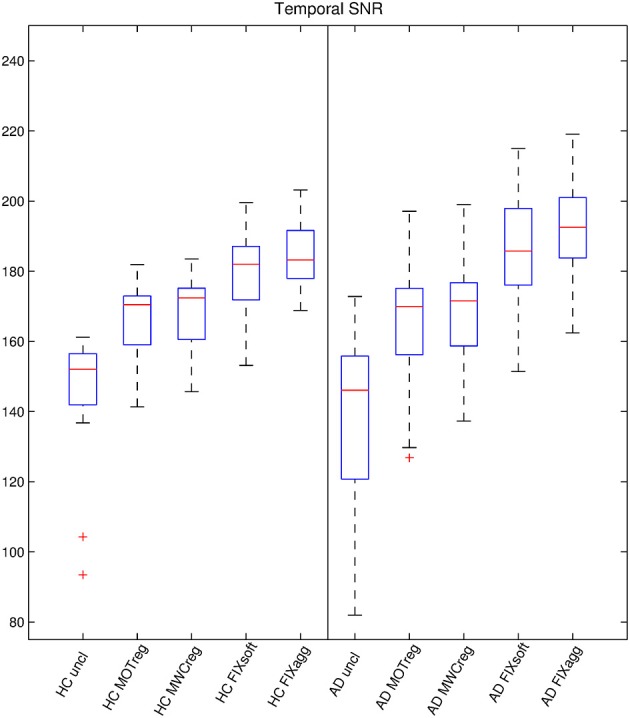
**Temporal SNR estimation**. For each subject and cleaning approach, a temporal-SNR image was formed dividing the mean image across time by the standard deviation image over time. The temporal-SNR image was then eroded to exclude brain-edge effects and the median value across space was calculated to represent the temporal SNR-value. The boxplots show the temporal SNR-value across subjects for various cleaning procedures in the two groups.

The temporal SNR-values for the two groups with different cleaning options are reported in Figure 1 and Supplementary Table 1, while the results of the comparisons across cleanings are shown in Supplementary Table 2. Within-group analyses revealed that SNR was significantly higher after cleaning (uncleaned < motion reg < MWCreg < FIX soft < FIX aggressive; *p* < 0.01, paired *t*-tests). The temporal SNR was not statistically different between the two groups, except for aggressive FIX clean-up (AD > HC, *p* = 0.044; independent two sample *t*-test).

**Figure 2:**
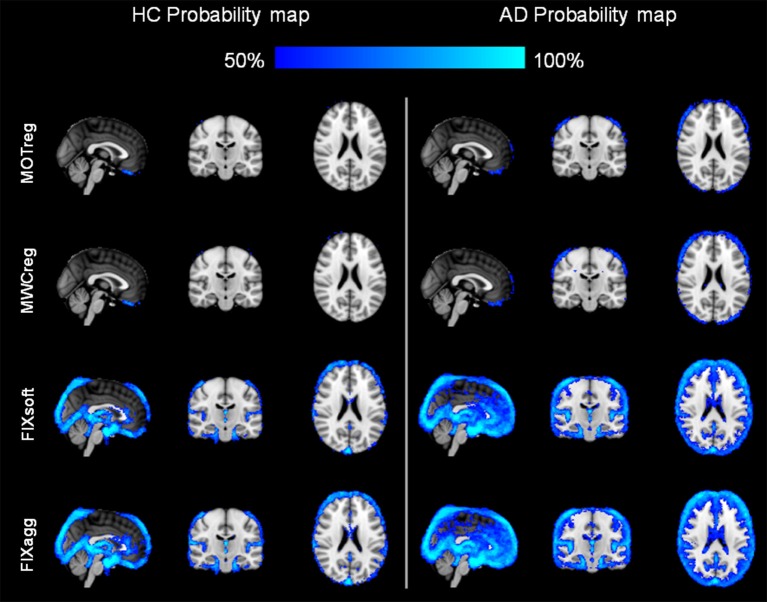
**Spatial pattern of changes in BOLD signal standard deviation**. A %ΔSTD map was created for each subject and cleaning approach using Equation 1 (see main text). The probability maps for each group and cleaning approach where then created by thresholding the single-subject maps to obtain the areas where %ΔSTD > 25%, registering the obtained maps to MNI standard space and calculating for each voxel the percentage of subjects with %ΔSTD > 25% separately for HC (**left**) and AD patients (**right**). Images are shown in radiological convention. (MNI coordinates *x* = 0, *y* = −18, *z* = 22).

The probability maps of the spatial distribution of BOLD fluctuation reduction (%ΔSTD > 25%) across subjects in the two groups are illustrated in Figure 2. After regressing out the contribution of motion parameters, the highest reduction of BOLD fluctuation was localized at brain boundaries, while the inclusion of WM and CSF regressors led to a small further decrease also in correspondence of the ventricles and the WM, especially in the AD group. After FIX clean-up (both soft and aggressive), the highest reduction of BOLD fluctuation with respect to uncleaned data was more pronounced at brain boundaries and within ventricles, but also involved the lateral sulcus and areas corresponding to blood vessels, mainly the sagittal sinus and straight sinus veins, the posterior cerebral artery and the middle cerebral branches. These effects were always higher in AD patients than HC.

### Within-group consistency results

**Figure 3:**
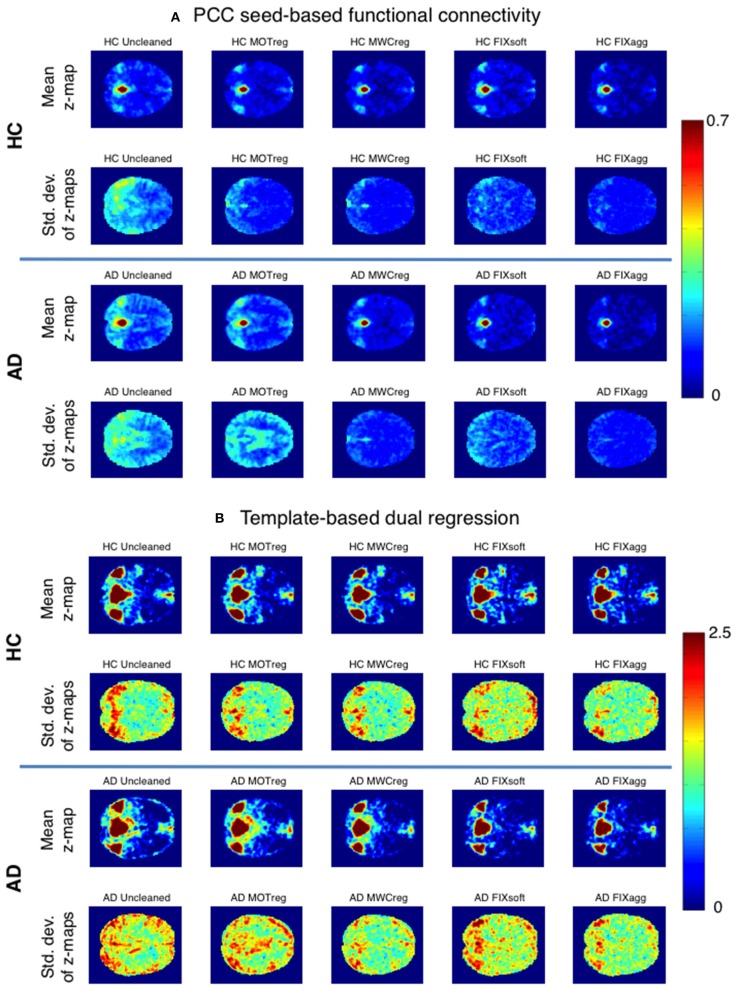
**Within-group consistency of PCC seed-based FC (A) and the DMN component obtained with template-based dual regression (B)**. A representative slice of the mean z-map (first and third row) and the standard deviation of z-maps (second and fourth row) across subjects is reported for HC (first and second row) and AD group (third and fourth row) after the different cleaning procedures.

The within-group consistency map (standard deviation across subjects of the z-maps obtained with seed-based correlation or template-based dual regression) for the two groups with different cleaning approaches is shown in Figure 3 (the relative mean maps across subjects are also reported). After all clean-up procedures we obtained higher within-group consistency across subjects both for seed-based and for template-based dual regression results, with the lowest standard deviation values across subjects being the ones obtained after MWCreg and FIXagg.

**Figure 4:**
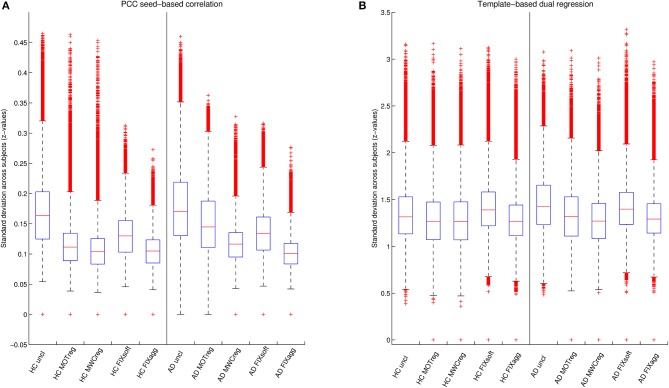
**Variability of DMN functional connectivity across subjects**. Standard deviation of *z*-values across space obtained with seed-based correlation **(A)** or template-based dual regression **(B)** on data cleaned with different cleaning procedures for the two groups (HC and AD).

The standard deviation *z*-values across space are showed in the boxplots in Figure 4 and the results of the statistical comparisons across cleanings are reported in Supplementary Table 3. In both groups the consistency increased significantly after cleaning (lower standard deviation) and the lowest standard deviation values were obtained after MWCreg and FIXagg. The standard deviation was, in general, lower (i.e., higher consistency) within the HC group (*p* < 0.01 at paired *t*-test) than in the AD group (except for seed based FC on FIXagg data and template-based dual regression MWCreg data, where the standard deviation was higher in HC than AD). Comparing the results across the FC analysis methods, the seed-based FC results showed a lower variability across subjects with respect to template-based dual regression, but also the mean z-statistics of the FC maps were much lower.

### Between-group differences in FC analysis

The results of the ROI analysis are shown in Table 2. The average P.E. within the PCC-precuneus was significantly lower in the AD group only after FIX agg cleaning both using seed-based analysis and template-based dual regression. A decreased FC in the AD is observable already after MWCreg and FIXsoft, but the between-group difference is not significant.

**Table 2:** **ROI analysis results**.

		**Parameter Estimates (P.E.)**		**Between-groups difference**	
		**Mean**	***Std. dev*.**	***t***	**Sig. (two-tailed)**
**SEED-BASED FC ANALYSIS**					
Uncleaned	HC	0.280	0.195	−0.542	0.591
	AD	0.314	0.208		
MOTreg	HC	0.203	0.084	−0.846	0.404
	AD	0.233	0.139		
MWCreg	HC	0.190	0.068	0.186	0.854
	AD	0.186	0.086		
FIXsoft	HC	0.222	0.080	1.525	0.135
	AD	0.177	0.107		
FIXagg	HC	0.175	0.062	2.327	0.025^*^
	AD	0.133	0.054		
**TEMPLATE-BASED DUAL REGRESSION**					
Uncleaned	HC	17.640	10.337	−0.556	0.581
	AD	19.588	11.971		
MOTreg	HC	12.296	4.089	0.049	0.961
	AD	12.222	5.332		
MWCreg	HC	11.712	3.471	1.011	0.318
	AD	10.619	3.446		
FIXsoft	HC	12.223	3.194	1.768	0.085
	AD	10.109	4.345		
FIXagg	HC	10.639	2.493	3.216	0.003^**^
	AD	8.117	2.527		

*Average P.E. in the PCC-Precuneus ROIs extracted from single subject maps obtained from data cleaned with different options using Seed-based FC or template-based dual regression analysis methods*.

* p < 0.05;

** *p < 0.01*.

**Figure 5:**
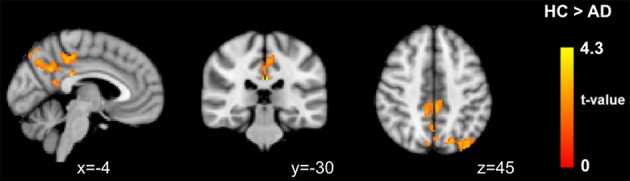
**Between-group differences in functional connectivity results using DMN template-based dual regression on data cleaned with FIX aggressive clean-up**. Images are shown in radiological convention.

Regarding the voxel-wise whole brain analysis, we observed no between-group differences at a corrected threshold with uncleaned data, MOTreg, MWCreg, and FIXsoft data with both seed-based FC and template-based dual regression. Only after FIXagg and using template-based dual regression approach we observed decreased FC in the AD group with respect to HC in the PCC, precuneus, and left superior and inferior parietal lobule (see Figure 5). Seed-based FC results with FIX aggressive showed a trend of significance (*p*_corr_ = 0.08) in PCC, precuneus, left middle and inferior temporal gyri, and left medial temporal lobe structures.

## Discussion

In this work we compared different approaches for the cleaning of rfMRI data in a group of elderly HC and a group of AD patients in mild stage of the disease in order to evaluate the impact of artifact removal in within-group and between-group analyses. The well-known FC alteration in the DMN was chosen as ground truth for “correct” functional connectivity estimation to compare the cleaning methods. Moreover, given the promising role of rfMRI as a biomarker in AD, the performance of the cleaning approaches was tested on data acquired using a clinical scanner at 1.5 T, as a test for translation in clinical practice.

We evaluated the impact of the different cleaning approaches on the BOLD signal variation in terms of temporal SNR and of percentage reduction of cleaned signal standard deviation with respect to the uncleaned data (%ΔSTD). The results showed an increase of SNR after each cleaning step and a localization of the reduction in BOLD signal fluctuation (%ΔSTD) in line with the well-known spatial characteristics of the artifacts being removed. In particular, the higher probability of BOLD fluctuation reduction after MOTreg is clearly observable at brain boundaries, where the motion-related artifacts are usually localized. This effect is more pronounced in the AD group, which showed higher mean relative displacement than the HC group, although not statistically significant. When FIX clean-up is performed, a consistent reduction of BOLD fluctuation is also localized in blood vessels and CSF. This suggests that this data-driven method is able to capture and remove also the physiological noise (vascular and CSF pulsation artifacts) in absence of external recordings. The higher reduction of BOLD fluctuations observed in AD patients with respect to HC is mainly localized in the periventricular areas and cortical sulci, regions that are more extended in AD, due to atrophy, as confirmed by the VBM analysis consistent with previous literature (Busatto et al., 2008; Zamboni et al., 2013).

Regarding the FC results, we observed that after all the clean-up procedures we obtained higher within-group consistency across subjects both for seed-based and for template-based dual regression results, but different results when evaluating between-group differences. After MOTreg no differences in FC were detected with both seed-based correlation and template-based dual regression, demonstrating that the removal of only the motion parameters is often not sufficient to perform an effective FC analysis. The lowest within-group standard deviation values across subjects in FC maps were obtained after MWCreg and FIXagg, and the spatial pattern was very similar. However, only after FIXagg the DMN alterations in AD patients were significantly detectable. Although both approaches remove the full variance of the artifacts and make the data more consistent across subjects, the removed signal is different: the WM and CSF components identified and removed by FIXagg are subject-specific and obtained from ICA decomposition, while the WM and CSF regressors removed in MWCreg are derived on an anatomical basis and could be affected by registration errors. Moreover, the localization of ROIs for MWCreg is an issue under debate (Chao-Gan and Yu-Feng, 2010), as they might contain some useful signal that is removed with the clean-up. Moreover, FIXagg removes other important confounds as vascular and susceptibility artifacts. These results demonstrate that a single measure of within-group consistency is not always sufficient to have a reliable measure of effectiveness of a cleaning procedure, because it is possible that useful across-subject variability, necessary to discriminate the two groups, is removed with the cleaning or that the cleaning is not effective enough to capture the between-group differences.

Compared to the most commonly used approaches for confounds removal, we showed that FIX was more effective in removing multiple sources of artifacts, and allowed the detection of pathological FC alterations. However, we acknowledge that the negative results (i.e., no significant between-group difference) obtained with the other methods are not to be considered in absolute terms as bad performance of other cleaning approaches. Increasing the sample size, the alteration of the DMN would probably become detectable with the other methods as well, and future studies being able to evaluate smaller between-approach differences in a larger sample will be certainly important. The fact that with such a small sample size we can see this alteration makes FIX the most sensitive approach.

Interestingly, although the alteration of the FC pattern was similar, the results were not significant at a corrected threshold with FIXsoft, but only with FIXagg. The soft and aggressive FIX options remove the same number of single-subject ICs, but with FIXagg the shared variance between the good and the artifactual components is also removed. We therefore hypothesize that this amount of variance could contain more artifacts, possibly related to morphological changes due to atrophy, and its removal in this specific target population is particularly beneficial. However, this hypothesis should be confirmed with future studies, for example using different scanners and sequences and in a bigger sample size.

It must be taken into account that, even if FIX approach was demonstrated to be the best option for detecting FC alterations in AD, a loss of meaningful and disease-specific information cannot be excluded. For example, there could be loss of neural-related signal that correlates with motion and with the vascular components. While no certain way for distinguishing neural-related signal correlating with motion parameters from real motion artifacts is known so far, and we must consider this possible loss as part of the optimized trade-off between noise removal and signal loss, interesting future development would be the specific study of the cardiovascular components in HC and AD and what is the clinical value of their neural and/or vascular related information.

We based our evaluation of FIX efficacy in AD on the assumption that FC in the DMN is significantly altered in AD with respect to HC even from the early stages of the pathology (Filippini et al., 2009; Sorg et al., 2009; Gili et al., 2011; Hafkemeijer et al., 2012; Cha et al., 2013; Esposito et al., 2013; Wang et al., 2013), since many studies have showed with other imaging modalities that DMN structures, involved in the memory processes, are particularly vulnerable to atrophy and to amyloid protein deposition, and usually show a reduced glucose metabolism (Minoshima et al., 1997; Buckner et al., 2005). It would be undoubtedly a corroboration of our findings to be able to replicate the differences we observed across cleaning modalities in a different population, with a different well-documented functional alteration.

The significant FC results obtained with seed-based correlation and template-based dual regression are consistent, but not identical, as the FC was measured in different ways: the seed-based connectivity results relate to the correlation of the mean signal within the PCC with all brain voxels, while the template-based dual regression approach evaluates the whole DMN connectivity pattern and with increased specificity coming from the inclusion (in the multiple regression) of other major networks. With ROI analysis, both methods were able to correctly detect the typical DMN alteration in AD patients, which involves the PCC and the precuneus (Greicius et al., 2004; Wang et al., 2007; Zhang et al., 2009; Gili et al., 2011; Binnewijzend et al., 2012). The voxel-wise analysis with template-based dual regression showed that the reduced FC is also extending toward the parietal cortex (Greicius et al., 2004; Wang et al., 2007). Although the ROI analysis results are similar with the two methods, the voxel-wise seed-based FC results were not significant. This is probably due to the small sample size and possibly by the fact that with seed-based correlation we were investigating the FC of a more localized area, as we wanted to answer a specific question, arising from previous literature evidence. As already pointed out by Cole and colleagues (Cole et al., 2010), it is advisable to use seed-based FC methods only under precise *a priori* hypothesis (e.g., in this case the PCC connectivity alteration), to ask a straightforward question about the FC of a specific area with the rest of the brain, to receive a direct answer. On the other hand, a data-driven approach, like group ICA or template-based dual regression, allows an overall view of the FC of the whole network of interest and allows the study of more than one RSN at the same time. Thus, this option is more advisable in absence of a precise hypothesis to test.

## Conclusion

In this work we compared four data-driven cleaning approaches on elderly HC and people with AD. We demonstrated the importance of an effective cleaning of rfMRI data of different sources of artifacts, in order to correctly detect FC alterations in this neurodegenerative condition even in the early stages of the disease, and even on data acquired using a clinical scanner at 1.5 T, as in our sample. These results obtained in a relatively small sample are promising results toward the definition of a reliable non-invasive biomarker for AD, as well as an instrument to monitor the staging of the disease.

### Conflict of interest statement

The authors declare that the research was conducted in the absence of any commercial or financial relationships that could be construed as a potential conflict of interest.
