# Supplementary material for: Effective artifact removal in resting state fMRI data improves detection of DMN functional connectivity alteration in Alzheimer's disease
Source: Front Hum Neurosci. 2015 Aug 11;9:449. doi: 10.3389/fnhum.2015.00449 (PMC4531245; doi:10.3389/fnhum.2015.00449)
Supplement: Supplementary file 1 [file Table1.DOCX]

***Supplementary Material***

**Effective artifact removal in resting state fMRI data improves detection of DMN functional connectivity alteration in Alzheimer’s disease**

**Ludovica Griffanti^1,2,3*^, Ottavia Dipasquale^1,2^, Maria M. Laganà^1^, Raffaello Nemni^1,4^, Mario Clerici^1,4^, Stephen M. Smith^3^, Giuseppe Baselli^2^, and Francesca Baglio^1^**

*^1^IRCCS, Fondazione don Carlo Gnocchi, Milan, Italy*

*^2^Department of Electronics, Information and Bioengineering, Politecnico di Milano, Milan, Italy*

*^3^FMRIB (Oxford University Centre for Functional MRI of the Brain), UK*

*^4^Physiopatholgy Department, Università degli Studi di Milano, Milan, Italy*

***Corresponding author**

Ludovica Griffanti

Oxford Centre for Functional MRI of the Brain (FMRIB)

Nuffield Department of Clinical Neurosciences, University of Oxford

John Radcliffe Hospital, Headley Way, Oxford, OX3 9DU, UK

e-mail: [ludovica.griffanti@ndcn.ox.ac.uk](mailto:ludovica.griffanti@ndcn.ox.ac.uk)

Phone number: +44 (0)1865 857671

Fax number: +44 (0)1865 222717

**1. Supplementary Figures and Tables**

**1.1. Supplementary Figures**

**Supplementary Figure 1**. **VBM results from group comparison.** Results are shown using a significance threshold of p<0.005 fully-corrected for multiple comparisons using threshold free cluster enhancement. Images are shown in radiological convention.

**1.2. Supplementary Tables**

**Supplementary Table 1**. **Temporal SNR (tSNR) values for different cleaning approaches.** The tSNR weighted for the number of timepoints is also reported for better comparison across studies.

|  | **HC** | | **AD** | |
| --- | --- | --- | --- | --- |
| **tSNR** | **Mean** | **Std.dev.** | **Mean** | **Std.dev.** |
| Uncleaned | 145.95 | 17.64 | 139.35 | 22.96 |
| MOTreg | 166.33 | 10.58 | 164.43 | 17.04 |
| MWCreg | 168.54 | 9.91 | 167.34 | 15.21 |
| FIXsoft | 179.52 | 11.58 | 186.50 | 15.12 |
| FIXagg | 184.75 | 9.93 | 192.98 | 14.73 |
|  |  |  |  |  |
| **tSNR*sqrt(timepoints)** | |  |  |  |
| Uncleaned | 1846.13 | 223.15 | 1762.62 | 290.42 |
| MOTreg | 2103.97 | 133.82 | 2079.89 | 215.56 |
| MWCreg | 2131.83 | 125.30 | 2116.74 | 192.37 |
| FIXsoft | 2270.80 | 146.42 | 2359.01 | 191.22 |
| FIXagg | 2336.89 | 125.67 | 2440.97 | 186.34 |

**Supplementary Table 2. Comparison of tSNR among different cleaning approaches (paired t-test).**

|  | **HC (t-values)** | **AD (t-values)** |
| --- | --- | --- |
| Uncleaned-MOTreg | -9.80 | -10.55 |
| Uncleaned-MWCreg | -9.51 | -10.30 |
| Uncleaned-FIXsoft | -12.86 | -10.27 |
| Uncleaned-FIXagg | -10.00 | -9.66 |
| MOTreg-MWCreg | -7.21 | -5.48 |
| MOTreg-FIXsoft | -11.38 | -7.86 |
| MOTreg-FIXagg | -9.26 | -8.30 |
| MWCreg-FIXsoft | -10.14 | -7.94 |
| MWCreg-FIXagg | -9.38 | -8.45 |
| FIXsoft-FIXagg | -3.62 | -3.31 |

All comparisons were statistically significant (p<0.01).

**Supplementary Table 3. Across-subjects standard deviation.** Comparison among different cleaning approaches in the two groups (paired t-test on the z-values of the standard deviation maps).

|  | **PCC seed** | | **template-based**  **dual regression** | |
| --- | --- | --- | --- | --- |
|  | **HC**  **(t-values)** | **AD**  **(t-values)** | **HC**  **(t-values)** | **AD**  **(t-values)** |
| Uncleaned-MOTreg | 511.3588 | 260.2754 | 137.3739 | 171.8571 |
| Uncleaned-MWCreg | 520.7048 | 478.4355 | 135.653 | 221.4 |
| Uncleaned-FIXsoft | 363.0041 | 363.8185 | -94.8889 | 53.7983 |
| Uncleaned-FIXagg | 524.6706 | 559.3019 | 98.5434 | 210.6886 |
| MOTreg-MWCreg | 276.2291 | 393.0219 | -3.4958 | 164.191 |
| MOTreg-FIXsoft | -191.9647 | 178.8484 | -194.3158 | -154.9582 |
| MOTreg-FIXagg | 147.2746 | 463.0707 | -81.4483 | -55.2723 |
| MWCreg-FIXsoft | -292.9114 | -264.4339 | -192.9672 | -210.0587 |
| MWCreg-FIXagg | 38.3945 | 249.9889 | -80.7162 | -107.1374 |
| FIXsoft-FIXagg | 502.2432 | 602.6446 | 638.9623 | 721.0622 |

All comparisons were statistically significant (p<0.01).
