# Supplementary figures and images for: Effective artifact removal in resting state fMRI data improves detection of DMN functional connectivity alteration in Alzheimer's disease
Source: Front Hum Neurosci. 2015 Aug 11;9:449. doi: 10.3389/fnhum.2015.00449 (PMC4531245; doi:10.3389/fnhum.2015.00449)

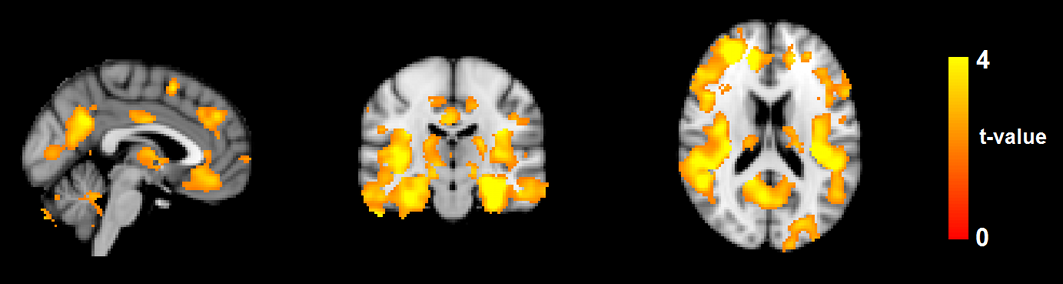

Supplement: Supplementary file 2 [file Image1.TIF]
